# Case report of neurobrucellosis: a rare complication and neuroimaging findings of a common disease

**DOI:** 10.3389/fimmu.2024.1449909

**Published:** 2025-01-07

**Authors:** Yu Zhang, Xiao-Yi Zou, Ling Liu

**Affiliations:** ^1^ Department of Neurology, West China Hospital, Sichuan University, Chengdu, Sichuan, China; ^2^ Department of Neurology, Chengdu Shangjin Nanfu Hospital, Chengdu, Sichuan, China

**Keywords:** neurobrucellosis, clinical manifestation, laboratory investigation, radiological feature, antibiotics therapy

## Abstract

**Background and objective:**

Neurobrucellosis is a rare neurological disorder characterized by diverse clinical manifestations. Although several relevant cases were reported, our understanding of this disorder is limited. In this study, we presented the clinical and imaging characteristics of four cases of neurobrucellosis.

**Methods:**

Four patients with neurobrucellosis were diagnosed and treated in the West China Hospital of Sichuan University and Chengdu Shangjin Nanfu Hospital, from January 2020 to September 2023. Data on demographics, clinical phenotypes and symptoms, cerebrospinal fluid investigations, radiological investigations, and therapies were collected and reviewed. This study was approved by and conducted in accordance with the recommendations of West China Hospital’s ethics -.1clinical manifestations of neurobrucellosis in these patients included meningitis, meningoencephalitis, encephalitis, cranial neuropathy, intracranial hypertension, radiculitis, peripheral neuropathy, myelitis, and other psychiatric symptoms. *Brucella* species were isolated from blood or cerebrospinal fluid (CSF) in four patients; three patients had elevated CSF protein levels, and two had elevated CSF leukocyte counts. All four patients had abnormal imaging findings, including meningeal signs, abnormal cortex and subcortical white matter signals, and signal abnormalities in the vertebral body and spinal cord. All patients were treated with rifampicin (450 mg once daily) and minocycline (100 mg twice daily) for at least 12 weeks, and their clinical symptoms showed significant improvements.

**Conclusion:**

This report reviews four cases of neurobrucellosis. All four patients had headache, fever, seizure, cranial nerve damage, low back pain, along with imaging abnormalities, and were successfully treated with antibiotics. The symptoms of neurobrucellosis can be insidious, mild, and non-specific, characterized by various clinical manifestations and atypical imaging findings. This complexity increases the risk of misdiagnosis and missed diagnosis; thus, careful identification, extended treatment, and close follow-up are required.

## Introduction

Human brucellosis is the most common zoonotic infection and is caused by any of the three main species of the genus *Brucella*, namely, *B. melitensis*, *B. suis*, and *B. abortus*. Most cases worldwide are caused by *B. melitensis* (1). Neurobrucellosis is an uncommon but serious complication of brucellosis (2). First described by Hughes in 1896, neurobrucellosis occurs in approximately 3%–5% of all brucellosis cases (3).

Neurobrucellosis has a wide range of neurological manifestations and may manifest either as part of systemic brucellosis or as an isolated disease (3). Neurological manifestations include acute meningitis or meningoencephalitis, cranial nerve involvement, myelitis, brain abscess, radiculitis, cerebellar involvement, subarachnoid hemorrhage, and psychiatric manifestations (4). The most common symptom of neurobrucellosis is headache, followed by blurred vision, hearing loss, confusion, sleep disturbances, epilepsy, agitation, depression, and manifestations of peripheral nerve involvement (4). Among the cranial nerves, the abducens, facial, and vestibulocochlear nerves are most frequently affected (5). Neurobrucellosis is typically diagnosed 2–12 months after symptom onset, and the disease may be insidious, with atypical clinical symptoms, often leading to a delayed diagnosis or misdiagnosis. As neurological complications can develop chronically, they are frequently misdiagnosed as other infections, such as tuberculosis or cryptococcal infection. They may also be misdiagnosed as demyelinating diseases such as multiple sclerosis or acute disseminated encephalomyelitis (6–8).

*Brucella* can effectively evade immune responses, easily spread throughout the body, and are difficult to eradicate with ordinary drugs, making it very difficult to cure (2, 6). To overcome this, drugs should be administered over an adequately long period, and a combination of multiple routes during acute treatment should be performed. Sufficient antimicrobial blood-brain barrier penetration is also required (2). Brucellosis is usually treated with a combination of three of the following antibiotics: ceftriaxone, rifampin, doxycycline, rifampin, and trimethoprim/sulfamethoxazole (2). It has a high relapse rate of approximately 5%–15%. Thus, patients must be followed up every 3 months, and treatment is usually continued for 6 months. Therapeutic failures are usually due to an insufficient duration of antibiotic treatment (2). Furthermore, drug resistance with antibiotic use remains a major concern. A study in Kazakhstan found that only 37.4% of *Brucella* bacteria strains were susceptible to rifampin, and similar resistance patterns were observed in a study in Iran (9, 10).

The nature of these lesions is not completely understood, but possible explanations are direct bacterial invasion or an autoimmune reaction (7, 8). To improve the ability of clinicians to identify neurobrucellosis and use specific antibiotic treatments as early as possible, we performed a retrospective analysis of the diagnosis and treatment process of four patients. This retrospective analysis was performed in accordance with the principles of informed consent. All patients provided written informed consent in accordance with the Declaration of Helsinki. This study strictly complies with the principles of voluntary participation and informed consent, ensuring that patients or their immediate family members, in cases where patients lacked self-awareness, were fully informed and voluntarily agreed to participate.

## Case presentation

### Case 1

#### Medical history

A 48-year-old male was referred to our hospital with severe headaches, bilateral hearing loss, and psychosis. His symptoms had started 6 months earlier with intermittent fever and headache accompanied by fatigue and asthenia. Over the course of 2 months, he developed arthralgia, osphyalgia, erythematous papular lesions, and psychosis. At a local hospital, magnetic resonance imaging (MRI) revealed signals in the right frontal cortex and subcortical white matter that were abnormally slightly thickened and strengthened near the meninges. Lumbar puncture revealed that the cerebrospinal fluid (CSF) protein level was 1.04 g/L (normal range, 0.15–0.45 g/L); leukocyte count, 41 ×10^6^/L; mononuclear cells, 82%; and glucose, 3.47 mmol/L (reference, 2.5–4.4 mmol/L). CSF Gram staining, acid-fast bacilli staining, and potassium hydroxide preparation results did not suggest a bacterial or fungal etiology. The patient was diagnosed with viral meningoencephalitis and underwent treatment with acyclovir and dexamethasone. After 10 days, his neurological symptoms did not improve, so he was referred to our hospital.

#### Physical and laboratory examinations

At our hospital, neurological examination revealed bilateral hearing loss and positive meningeal stimulation. Hearing examination suggested frequency-induced hearing loss at 1000–8000 Hz. The results of other examinations at this time were within normal limits. Lumbar puncture revealed clear CSF. The CSF leukocyte count was 81 ×10^6^/L (89% lymphocytes); CSF protein level, 1.36 g/L (normal range, 0.15–0.45 g/L); and glucose level, within normal range. The CSF was negative for oligoclonal bands. The CSF pathogen microbial-targeted gene surveillance result was positive (103 copies/mL), while the CSF cryptococcal antigen test and polymerase chain reaction for *Mycobacterium tuberculosis* yielded negative results. Blood and CSF cultures subsequently showed the growth of *Brucella* species (**Table 1**).

**Table 1:** Normal information and clinical data of death patients.

	Patient 1	Patient 2	Patient 3	Patient 4
Gender	Male	Male	Male	Male
Age(year)	48	33	38	26
Profession	Feeding sheep	Selling cattle and sheep	Unspecified	Feeding sheep
Contact with domesticated animals	Being bitten by a sheep	cattle and sheep	Living in pastoral areas	sheep
Symptoms and signs	headache and bilateral hearing loss,erythematous papular lesions , arthralgia, fever, osphyalgia,psychiatric manifestations	Headache, fever,seizure	Waist, hip, and lower limb pain	Headache, seizure
Duration of symptoms (months)	6 months	15 days	2 years	60 days
MRI, abnormality	Abnormal signals in the right frontal cortex and subcortical white matter, slightly thickened and strengthened near the meninges	Positive, abnormal signals in bilateral frontal and temporal cortex	Positive,paravertebral body and L5 / S1 vertebral space-S1 vertebral body plane vertebral canal,with a heterogeneous enhancement	The left temporal lobe showed lamelate long T1 slightly longer T2 signal, slightly higher signal on FLAIR, no enhancement scan and significant thickening of adjacent meninges with enhancement
EEG, abnormality	positive	positive	–	negative
CSF pressure(mmH2O)	170/120	150/80	–	220/150
CSF leukocytes (10^6/l)	42	6	–	490
Lymphocyte predominance (%)	82	75	–	80
CSF protein (g/L)	1.04	0.96	–	2.27
CSF glucose (mmol/L)	3.42	2.98	–	3.78
CRP, mg/L	7.46	6.73	8.54	73.3
Serum Coombs-Wright	Positive	Positive	Positive	Positive
CSF Wright	Positive	negative	–	Positive
Blood culture	B.Brucella species	Brucella species	Brucella species	Brucella species
Pathology	Few lymphocytes were seen in CSF shed cells	Few lymphocytes were seen in CSF shed cells	Lumbar tissue microscopic examination reveals an abundance of acute and chronic inflammatory cells with tissue infiltration and cellular aggregation, along with granulation tissue proliferation	Few lymphocytes were seen in CSF shed cells
CSF culture	B.Brucella species	Brucella species	–	Brucella species
Mortality	No	No	No	No
Therapeutic regimen	Rifampicin( 900mg once daily), ceftriaxone 4 g/day and doxycycline (100mg twice daily)	Rifampicin( 900mg once daily), ceftriaxone 4 g/day and doxycycline (100mg twice daily)	Rifampicin( 900mg once daily), ceftriaxone 4 g/day and doxycycline (100mg twice daily)	Rifampicin( 900mg once daily), ceftriaxone 4 g/day and doxycycline (100mg twice daily)
At follow-up at 6 months	The patient was asymptomatic and blood cultures were negative,brain MRI lesions disappeared	The patient was asymptomatic and blood cultures were negative,brain MRI lesions disappeared	The patient was asymptomatic and blood cultures were negative, lumbar MRI lesions decreased	The patient was asymptomatic and blood cultures were negative,brain MRI lesions disappeared

#### Cranial MRI

**Figure 1:**
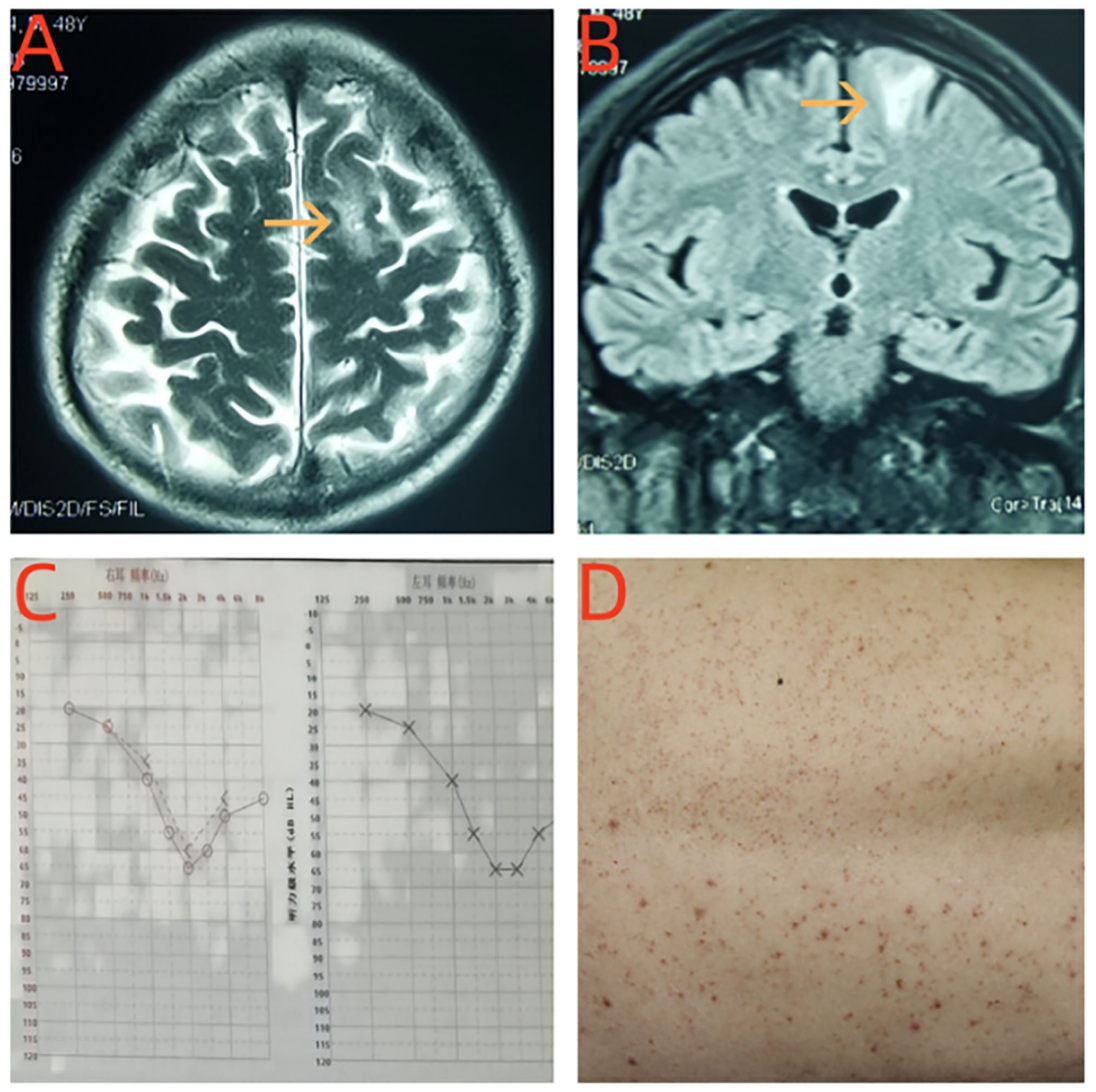
**(A, B)** Brain MRI revealed T2-weighted recovery signal hyperintensity in the right frontal lobe cortical and subcortical white matter abnormal signal. **(C)** The hearing examination of the patient showed moderate sensural sineural hearing loss in both ears. **(D)** The patient developed erythematous papular lesions on the skin of his back.

As with the local hospital MRI, enhanced brain MRI at our hospital revealed hyperintense lesions on T2WI involving the right frontal cortex and subcortical white matter that were abnormally slightly thickened and strengthened near the meninges (**Figure 1**).

#### Medication treatment

The patient also had a history of being bitten by sheep. He was treated with ceftriaxone, rifampicin (900 mg once daily), and doxycycline (100 mg twice daily) for 12 weeks. At the 6-mo follow-up, he was asymptomatic, blood cultures were negative, and the brain lesions seen on MRI disappeared.

### Case 2

#### Medical history

A 33-year-old male had developed headache and fever 15 days prior, mainly paroxysmal pain in the forehead with his body temperature peaking at 37.5 °C. There was no significant improvement after symptomatic treatment for analgesia and fever reduction. He experienced clonic and impaired awareness 10 days prior, which improved in approximately 10 minutes. This was accompanied by his eyes gaze, which was not preceded by aura. He did not experience tongue biting, urinary or fecal incontinence, salivation, or trauma. He had persistent headache with a body temperature of up to 40°C 1 day prior, accompanied by muscle soreness and general fatigue.

#### Physical and laboratory examinations

At the time of examination, he was conscious but ill, with a temperature of 38.5°C, respiratory rate of 20/minutes, pulse rate of 100/minutes, and blood pressure of 103/67 mmHg. His neurological examination was normal, and initial laboratory tests, including complete blood count, showed hemoglobin levels of 88 g/L, total leukocyte count of 3.04 ×10^9^/L, and platelet count of 72 ×10^9^/L. C-reactive protein (CRP) levels, erythrocyte sedimentation rate, and renal and liver function test results were within normal limits. Immunochromatographic tests for hepatitis B surface antigen, hepatitis C virus, and human immunodeficiency virus were negative, and a quantitative enzyme-linked immunosorbent assay for brucellosis demonstrated high levels of immunoglobulin (Ig) M and IgG for *B. abortus*. Blood culture revealed the presence of *Brucella*.

Lumbar puncture revealed clear CSF. The CSF leukocyte count was 6 ×10^6^/L (89% lymphocytes); CSF protein level, 0.96 g/L (normal range, 0.15–0.45 g/L); and glucose level, within normal range. Serum blood glucose was 5.4 mmol/L. The CSF was negative for oligoclonal bands (**Table 1**).

#### Cranial MRI

**Figure 2:**
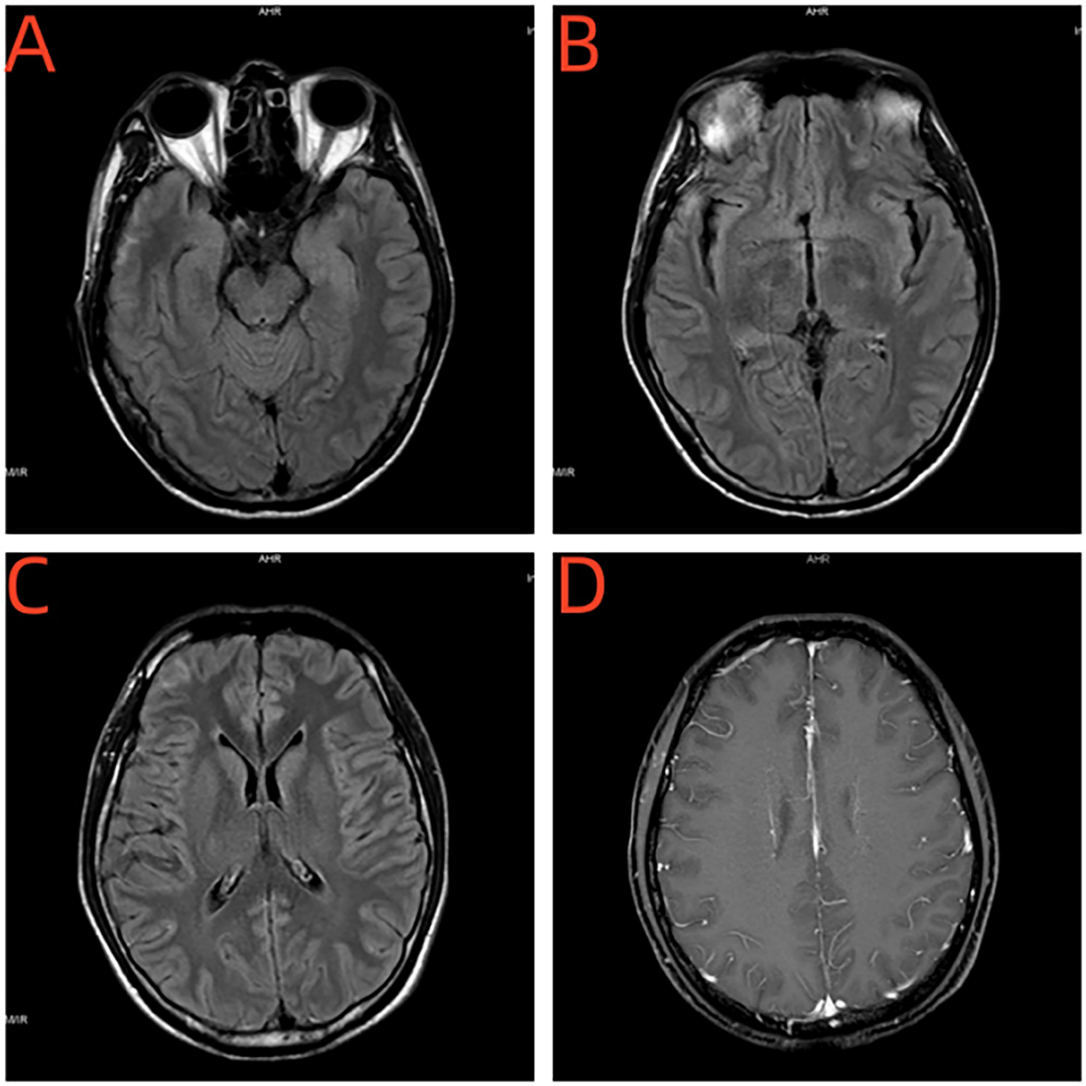
**(A–C)** Brain MRI revealed abnormal bilateral frontotemporal subcortical signals. **(D)** No enhancement was seen in the abnormal signal lesions.

Enhanced brain MRI revealed abnormal bilateral frontotemporal subcortical signals, no enhancement was seen in the abnormal signal lesions (**Figure 2**).

#### Medication treatment

The patient was treated with ceftriaxone, rifampicin (900 mg once daily), and doxycycline (100 mg twice daily) for 12 weeks. At a follow-up 12 weeks post-discharge, the patient was doing well and was compliant with medication.

### Case 3

#### Medical history

A 38-year-old male presented with pain in the lumbar and gluteal regions and both lower limbs for 2 years, with persistent attacks. Pain in the lumbar region resembled a pin prick, and pain in both lower limbs was accompanied by distension. The symptoms were often triggered by prolonged sitting, lying down, or walking and were relieved after changing positions. There were no other symptoms, such as difficulty in urination or defecation and numbness in the lower limbs, and neurological examination showed no special findings. The patient had been asked to live in a pastoral area for a long time prior to his symptom development.

#### Cranial MRI

**Figure 3:**
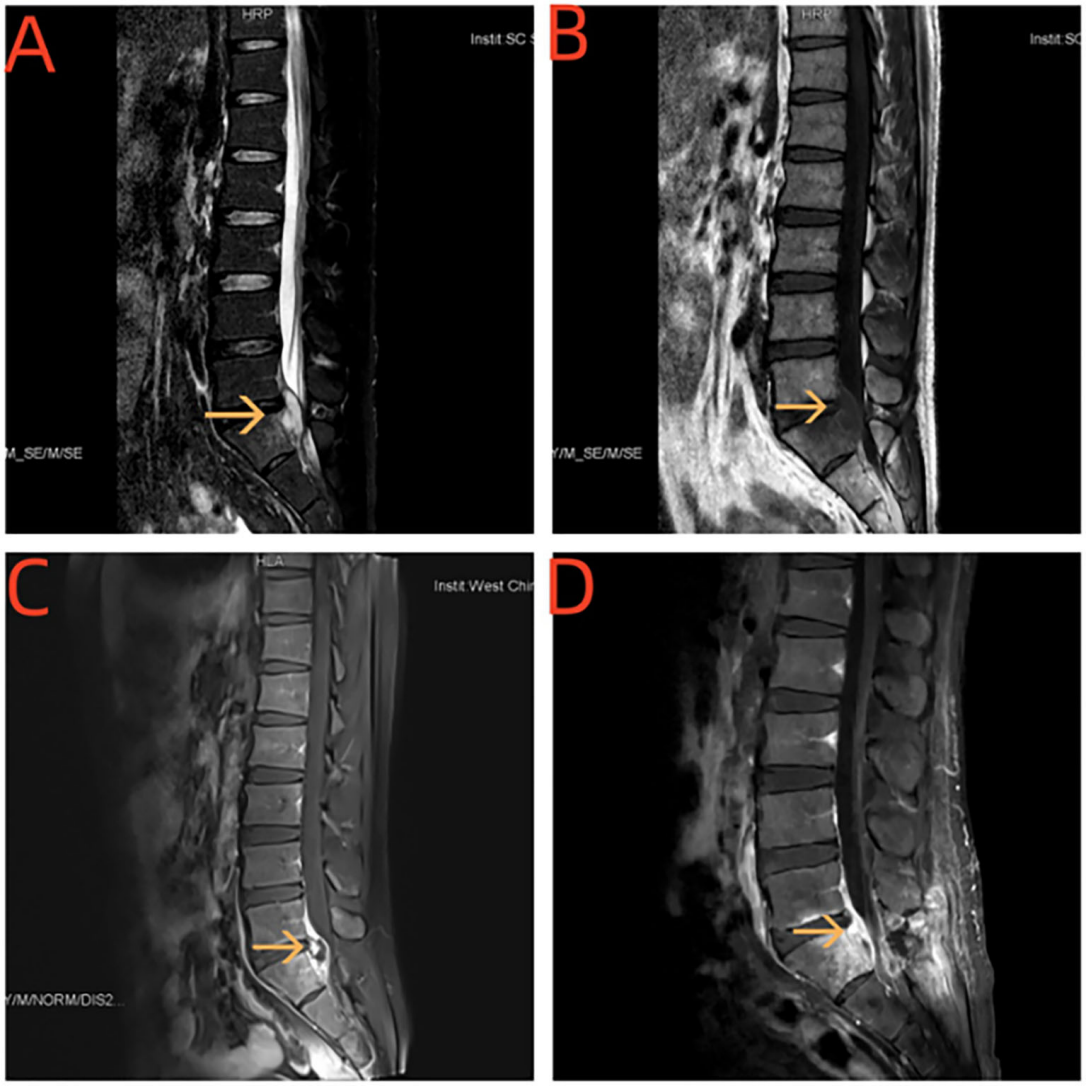
**(A, B)** MRI of the spinal cord showed paravertebral body and L5 / S1 vertebral space-S1 vertebral body plane vertebral canal see sheet long T1 long T2 signal shadow. **(C, D)** Enhancement and uneven enhancement class has spinal membrane thickening and enhancement.

Lumbar MRI revealed a lumbar 5-sacral 1 intraspinal space-occupying lesion; its nature was undetermined. After excluding contraindications related to surgery, lumbar 5-sacral 1 extradural space-occupying lesion resection, nerve root decompression, and sacral 1 lamina decompression were performed. He experienced significantly increased pain in the right hip and lower limbs 12 days post-surgery. Reexamination of the lumbar MRI showed a lack of bone in the S1 attachment, with swelling of the surrounding and lower lumbosacral soft tissue. We also found abnormal signals with significant narrowing of the canal at the paraspinal and L5/S1 intervertebral space-S1 vertebral anterior portion of the spinal canal and S1 and L5 vertebra. Compared to the previous MRI, the range of abnormal signals in the spinal canal was slightly increased, degree of swelling of the paraspinal soft tissue was increased, and new abnormal signals were detected in the L5 vertebral body (**Figure 3**).

#### Pathological and laboratory examinations

Microscopic examination revealed numerous acute and chronic inflammatory cells with tissue infiltration, tissue cell aggregation, and granulation tissue proliferation. The patient tested negative for cryptococcal, tuberculosis and fungal antibodies. Blood tests for *Brucella* antibodies were positive, and blood culture revealed *Brucella* (**Table 1**).

#### Medication treatment

He was treated with oral rifampicin (900 mg once daily) and oral doxycycline (100 mg twice daily; morning and evening) for 12 weeks. After 3 months, he returned for a follow-up examination and reported significant pain relief at his waist, hips, and lower limbs. MRI of his lumbar spine showed reduction in the lesions.

### Case 4

#### Medical history

A 26-year-old male presented with headache along with fever for 2 months and seizures for 2 days. He developed headache without obvious causes, mainly acute onset bilateral temporal pain accompanied by fever, with a maximum body temperature of 39.3°C. He showed no blurred vision, limb twitching, babbling, or consciousness disorders. He was treated at a local hospital and was considered to have “tuberculous meningitis” after treatment with rifampicin, isoniazid, ethambutol, and pyrazinamide. The symptoms did not improve significantly after treatment. He showed impaired awareness and limb tonic and clonic 2 days prior, which lasted for approximately 5 minutes before improving.

#### Physical and laboratory examinations

Neurological physical examination showed no abnormalities, and laboratory examinations were included that his C-reactive protein level was 73.30 mg/L, erythrocyte sedimentation rate was 54.0 mm/H, and tuberculosis and fungal tests were negative. Lumbar puncture revealed an initial pressure of 220 mmH_2_O and a terminal pressure of 150 mmH_2_O. Routine CSF analysis revealed the presence of 490 10^6^/L nucleated cells. CSF biochemical analysis showed 2.27 g/L of trace protein, and synchronous blood glucose was 5.79 mmol/L. The CSF was negative for oligoclonal bands. No bacteria, fungi, cryptococci, or mycobacteria were detected in the smears. *B. melitensis* was identified with a titer of 1:50 using CSF pathogen microbial-targeted gene sequencing (**Table 1**).

#### Cranial MRI

**Figure 4:**
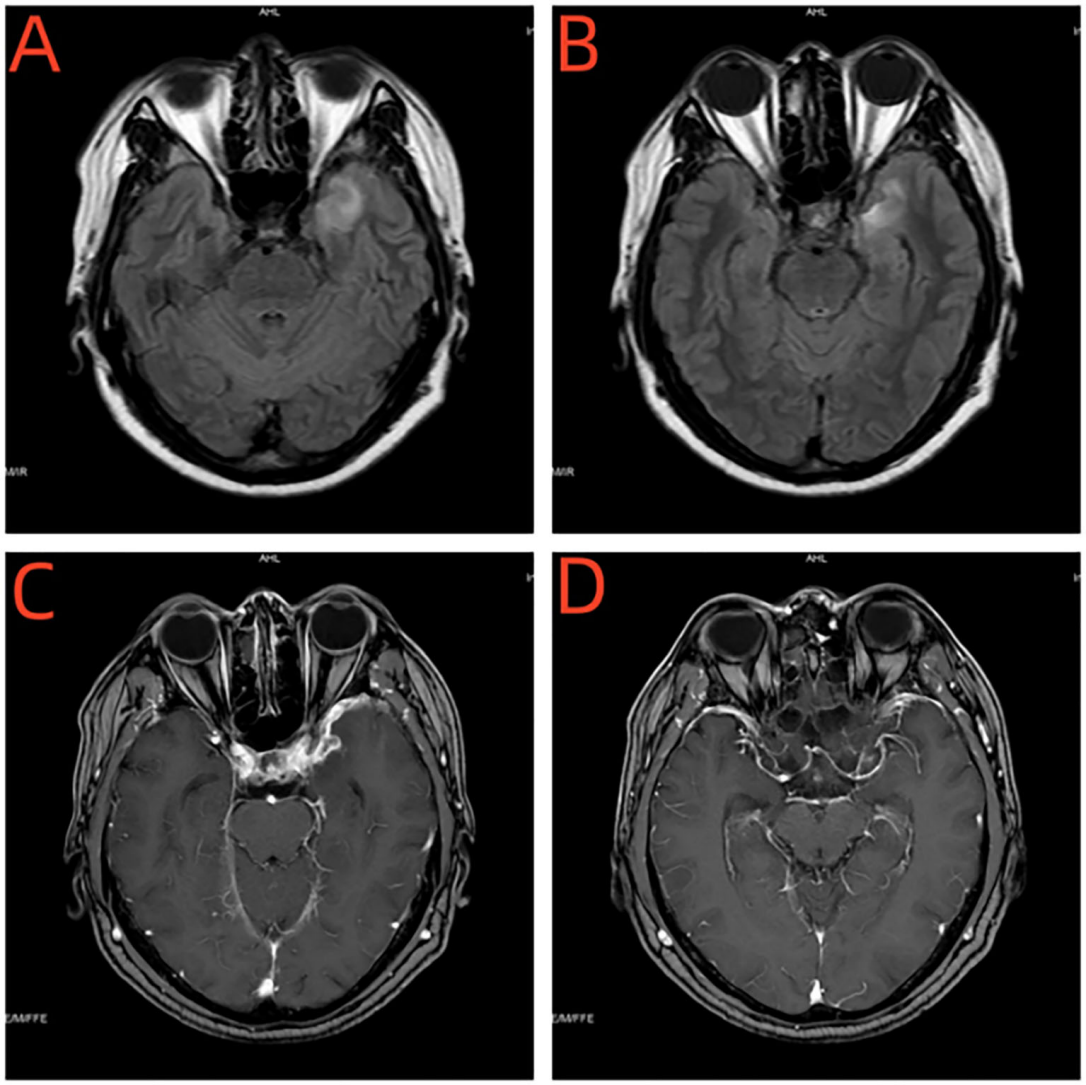
**(A, B)** Enhanced MRI of the head showed a sheet-like long T1 and slightly long T2 signal in the left temporal lobe, with a slightly higher signal on FLAIR. **(C, D)** Enhanced MRI of the head showed no significant enhancement on enhanced scan, and obvious thickening of adjacent meninges with enhancement.

Enhanced MRI of the head showed a sheet-like long T1 and slightly long T2 signal in the left temporal lobe, with a slightly higher signal on FLAIR, no significant enhancement on enhanced scan, and obvious thickening of adjacent meninges with enhancement (**Figure 4**).

#### Medication treatment

The patient was administered levetiracetam (500 mg twice daily), rifampicin (450 mg once daily), and minocycline (100 mg twice daily) for at least 12 weeks. After 3 months, he was followed up and showed significant improvement concerning his headache without further fever or epileptic seizures. After 6 months, follow-up MRI and electroencephalography showed no obvious abnormalities, and the remaining medication of levetiracetam was stopped.

## Discussion

Brucellosis is a zoonotic infectious disease caused by *Brucella*, which affects multiple systems in humans and animals (1). In China, sheep (infected by *B. melitensis*) are the most common source, followed by cattle (infected by *B. abortus*) and pigs (infected by *B. suis*) (6). The majority of infected individuals in China are middle-aged men between the ages of 41 to 65 years (6). This is largely because this demographic is the main labor force of their families and society and has more access to livestock (5). Neurological involvement is an important but rare complication of brucellosis, with less than 5% reported in patients (11). The clinical manifestations of neurobrucellosis are diverse, and the symptoms and signs lack specificity. Common symptoms include fever, fatigue, night sweats, joint and muscle pain, and liver and spleen enlargement (4). Neurological changes include meningitic changes, intracranial vascular injury, demyelinating lesions of the central nervous system, peripheral nerve lesions, and increased intracranial pressure (4). The involvement of nerves is commonly seen in damaged auditory, abductor, facial, and optic nerves (4). Specific manifestations include headache, neck stiffness, visual rotation, hearing loss, consciousness disorders, and limb weakness (4). Among the four patients included in this study, three presented with headache, which is consistent with the findings of previous reports identifying headache as the primary symptom. Two patients developed fever, and two patients experienced epilepsy. One patient exhibited erythematous papular lesions, arthralgia, hearing loss, and psychiatric symptoms. One patient primarily presented with spinal nerve root pain. These clinical manifestations are consistent with those reported in previous studies.

MRI findings of neurobrucellosis often present as abnormal enhancement of the meninges, which is more common at the bottom surface and is different from the convex surface seen in bacterial meningitis (12). The disease can also present as a granuloma, neuritis, or “ring target” sign in the brain parenchyma, hemorrhage, mild perivascular enhancement, and diffuse white matter changes (12). Demyelinating white matter lesions present as diffuse, periventricular, and focal T2 hyperintensities (12). Vascular lesions include: (1) inflammatory processes in small blood vessels or venous systems, causing lacunar infarction, minor hemorrhage, or venous thrombosis; (2) fungal arterial rupture that can cause hemorrhagic stroke; and (3) arterial involvement that can lead to transient ischemic attacks or ischemic stroke (12). In our study, two patients exhibited significant meningeal enhancement, and three patients exhibited abnormal signals in the brain parenchyma, consistent with the findings of previous reports. One patient presented with abnormal signals in the lumbar spinal cord, spinal membrane, nerve root, and vertebral body, a finding that was relatively rare in previous reports.

In 1963, Fincham et al. reported that the white matter changes in neurobrucellosis were sequelae of the demyelination, as confirmed by pathologic study (10). Marconi supported this with autopsy evidence of demyelination similar to multiple sclerosis lesions in one patient with neurobrucellosis (13). This suggests that white matter involvement is related to an immune-mediated reaction in the central nervous system to the Brucella infection. And the main features of the diffuse involvement of the cerebral white matter were astrogliosis and reactive microgliosis.

The pathogenesis of neurobrucellosis is complex, with varying degrees of of fungi, toxin, and allergen involvement. The following have been established: (1) brucellosis directly or indirectly invades the blood-brain barrier; (2) dysfunction of the blood-brain barrier; (3) activation of protein kinase pathways; (4) upregulation of innate immune receptors; and (5) demyelination of nerve cells and formation of ganglioside antibodies (14).

There are no specific criteria for the diagnosis of neurobrucellosis; rather, diagnosis is based on a compatible clinical picture, imaging abnormalities, and evidence of *Brucella* infection in the CSF or blood (15). Specifically, this includes the presence of neurological symptoms not explained by any other disease, isolation of *Brucella* in culture, positive serological tests in the blood or CSF, abnormal CSF parameters (cellularity, protein, and glucose), and clinical response after antibiotic treatment (16). Although bacterial culture is the gold standard for diagnosing infections, CSF culture is often negative in neurobrucellosis cases (5, 14, 16) and positive in only 12%–24% of patients (16). Therefore, neurobrucellosis is usually diagnosed by detecting *Brucella* antibodies in the serum or an increase in the *Brucella* antibody titers in the CSF using various agglutination tests. In our study, *Brucella* cultures were positive in serum and CSF in all four patients, which contrasts with the findings of previous reports. This discrepancy could be attributed to the small sample size and selection bias, as the patients included were all those with a definitive diagnosis. Thus, a large sample size is needed for further analysis and validation of our findings. Three of the four patients underwent CSF testing; three patients had elevated protein levels, and two patients had elevated leukocyte counts. However, the elevated CSF parameters are not characteristic of neurobrucellosis and can also be seen in other inflammatory conditions such as tuberculosis and viral encephalitis.

Due to the lack of clear specificity in the clinical symptoms of neurobrucellosis and the often-negative results of many laboratory tests commonly used to diagnose neurobrucellosis, there is no clear clinical diagnostic criteria or treatment for brucellosis. This leads to low clinical diagnosis and high misdiagnosis rates, and it is difficult to distinguish neurobrucellosis from many other neurological diseases, including tuberculous meningitis and multiple sclerosis (7, 17). When patients present with neurological dysfunction with fever and fatigue or have a history of contact with animals and animal products, the possibility of neurobrucellosis should be considered and diagnostic treatment for neurobrucellosis should be given if necessary.

Neurobrucellosis treatment involves a combination of antibiotics. The permeability of the blood-brain barrier should be considered in antibiotic selection. Therefore, third-generation cephalosporins (such as ceftriaxone and cefotaxime), rifampicin, and cotrimoxazole, which can penetrate the central nervous system, should be used in combination. Ceftriaxone (4 g/day) for the first 4–6 weeks, in addition to rifampin at 15 mg/kg/d (600–900 mg) and doxycycline (100 mg twice daily) for at least 12 weeks, is considered the first-line therapy (12). Relapses are common (5%–15% of cases) and frequently result from missed diagnoses of complications, poor compliance with a prolonged course of therapy, inappropriate antibiotic selection, or improper treatment of focal infection (12). In our study, all four patients were treated with rifampicin (450 mg once daily) and minocycline (100 mg twice daily) for at least 12 weeks. All patients adhered to their medication regimen, showed no signs of drug resistance, and experienced good therapeutic outcomes.

This paper discusses a series of only four cases; therefore, more studies are needed to further elucidate and validate the condition, indications, and appropriate treatment of neurobrucellosis.

## Conclusions

In our study, all four patients exhibited clinical manifestations, imaging characteristics, and CSF findings similar to those of chronic inflammatory conditions, which were not specific for neurobrucellosis. The diagnosis of neurobrucellosis mainly depends on the positive detection of *Brucella* antibodies in serum or CSF. In endemic areas, patients with severe and persistent headaches should be tested for neurobrucellosis. The treatment requires the combined use of any three aforementioned antibiotics at adequate doses for sufficient duration; otherwise, recurrence is likely.

## Data Availability

The datasets presented in this article are not readily available because of ethical and privacy restrictions. Requests to access the datasets should be directed to the corresponding author.
